# Prognostic value of coronary CTA-based classifications for predicting major events without obstructive coronary artery disease

**DOI:** 10.1038/s41598-023-37465-7

**Published:** 2023-06-30

**Authors:** Zengfa Huang, Beibei Cao, Xinyu Du, Mei Li, Jiong Huang, Zuoqin Li, Jianwei Xiao, Xiang Wang

**Affiliations:** 1 Department of Radiology, The Central Hospital of Wuhan, Tongji Medical College, Huazhong University of Science and Technology, 26 Shengli Avenue, Jiangan, Wuhan, 430014 Hubei China; 2 Department of Community Health, Hanyang District Center For Disease Control and Prevention, Wuhan, 430050 Hubei China; 3 Department of Radiology, The Central Hospital of Wuhan Base, Hubei University of Medicine, Shiyan, 442000 Hubei China; 4 Department of Radiology, The Sixth Hospital of Wuhan, Affiliated Hospital of Jianghan University, 168 HongKong Road, Jiangan, Wuhan, 430015 Hubei China

**Keywords:** Cardiology, Health care

## Abstract

We aim to explore the classifications based on coronary computed tomography angiography (CTA) for predicting the risk of major adverse cardiovascular events (MACE) in patients with suspected non-obstructive coronary artery disease (CAD) and compare with traditional non-obstructive CAD (NOCAD) classification, Duke prognostic NOCAD index, Non-obstructive coronary artery disease reporting and data system (NOCAD-RADS). 4378 consecutive non-obstructive CAD patients were assessed by coronary CTA for traditional NOCAD classification, Duke prognostic NOCAD index, NOCAD-RADS and a new classification (stenosis proximal involvement, SPI) from two medical centrals. We defined proximal involvement as any plaque was present in the main or proximal segments of coronary artery (left main, left anterior descending artery, left circumflex artery, or right coronary artery). The main outcome was MACE. During a median follow-up of 3.7 years, a total of 310 patients experienced MACE event. Kaplan–Meier survival curves showed the cumulative events increased significantly associated with traditional NOCAD, Duke NOCAD index, NOCAD-RADS and SPI classifications (all *P* < 0.001). In multivariate Cox regressions, the risk for the events increased from *HR* 1.20 (95% CI 0.78–1.83,* P* = 0.408) for SPI 1 to 1.35 (95% CI 1.05–1.73, *P* = 0.019) for SPI 2, using SPI 0 as the reference group. Coronary CTA based SPI classification provided important prognostic information for all cause-mortality risk and MACE prediction in patients with non-obstructive CAD, which was non-inferior than traditional NOCAD, Duke NOCAD Index and NOCAD-RADS classifications. The plaque location information by coronary CTA may provide additional risk prediction in patients with non-obstructive CAD.

## Introduction

Cardiovascular disease (CVD) is a worldwide disease and the major contributor to reduced quality of life^1^. CVD is also the leading cause of both mortality and premature mortality in China, accounting for 40% of the death in the Chinese population^2^. Historically, coronary artery disease (CAD) is defined as the presence of obstructive coronary artery stenosis (≥ 50%) in one or more coronary vessels and most of current prevention and treatment protocols are in accordance with this paradigm: removing the obstruction for treating angina and preventing myocardial infarction^3^. However, a recent research has demonstrated that approximately two-thirds of the patients were belong to without obstructive CAD in the CONFIRM (Coronary CT angiography evaluation for clinical outcomes: an international multicenter) registry study^4^. Moreover, a large prospective trial recent reported that the majority of cardiovascular events occurred among patients with non-obstructive CAD^5^.

Coronary computed tomography angiography (CTA) is a non-invasive imaging technique that allows for accurate detection and assessment of non-obstructive CAD^6^. One important feature of coronary CTA is that it provides information on the presence, location, and quantity of coronary atherosclerotic lesions^7,8^. The prognostic significance of the presence and stenosis degree of coronary atherosclerotic lesions by coronary CTA has been well established^9,10^. Furthermore, plaque location of coronary atherosclerotic lesions was integrated into a comprehensive CTA score and showed a good prediction of future events^8^. Moreover, acute coronary events in proximal vessels are more likely to lead to a clinically significant event as proximal vessels supply more myocardium. Previous studies have demonstrated that the proximally located plaque is associated with poor prognosis in obstructive CAD patients detected by coronary CTA^11^. In addition, only a few studies assessed the prognostic value of proximal plaque location with major adverse cardiovascular events (MACE) in non-obstructive CAD patients^12^. However, the contribution of proximal plaque location to MACE in non-obstructive CAD patients has not been studied in Chinese population. Moreover, the prognostic performance of proximal plaque location to MACE in non-obstructive CAD patients has not been compared with the existed classifications of non-obstructive CAD. Thus, we aim to investigate the classifications based on coronary CTA for predicting the risk of MACE in patients with suspected non-obstructive CAD and then compare with traditional non-obstructive CAD (NOCAD) classification, Duke prognostic NOCAD index, Non-obstructive coronary artery disease reporting and data system (NOCAD-RADS).

## Methods

This is a retrospective, observational, multicentre study. The trial protocol have been reviewed and approved by the ethics committee of the Central Hospital of Wuhan, Tongji Medical College, Huazhong University of Science and Technology and was conducted in compliance with the Health Insurance Portability and Accountability Act (HIPAA) of 1996. Written informed consent was waived because of its retrospective observational nature and waiver for informed consent is approved by ethics committee of the Central Hospital of Wuhan, Tongji Medical College, Huazhong University of Science and Technology.

### Study population

**Figure 1:**
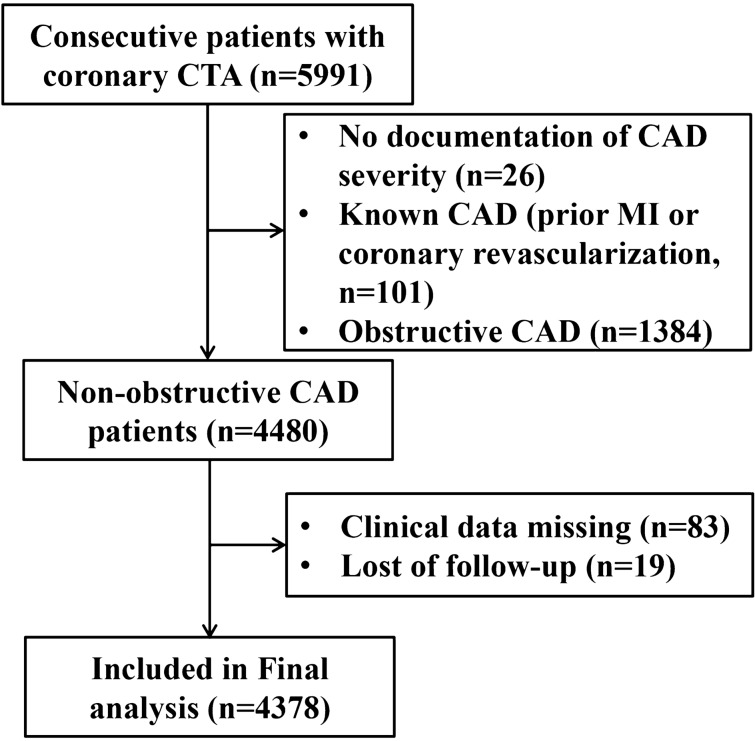
Flowchart of participant selection for analysis in the present study.

This study population consisted of 5991 consecutive patients with suspected CAD who were performed coronary CTA for clinical reasons between June 2017 and December 2019 at two hospitals in Wuhan, China. We have previous reported the details of the rational of the study and included parts of the patients^13^. We used the first coronary CTA examination to characterize CAD extent if multiple coronary CTAs were performed during the study period. In the current study, we excluded patients with no documentation of CAD severity (n = 26), prior history CAD or revascularization (defined as previous myocardial infarction, underwent percutaneous coronary intervention or coronary artery bypass grafting, n = 101), obstructive CAD (n = 1384), clinical data missing (n = 83) and loss of follow-up (n = 19) (Fig. 1). Finally, 4378 patients without obstructive CAD were included in the current analysis.

### Coronary CTA protocol and coronary CTA based classifications

**Figure 2:**
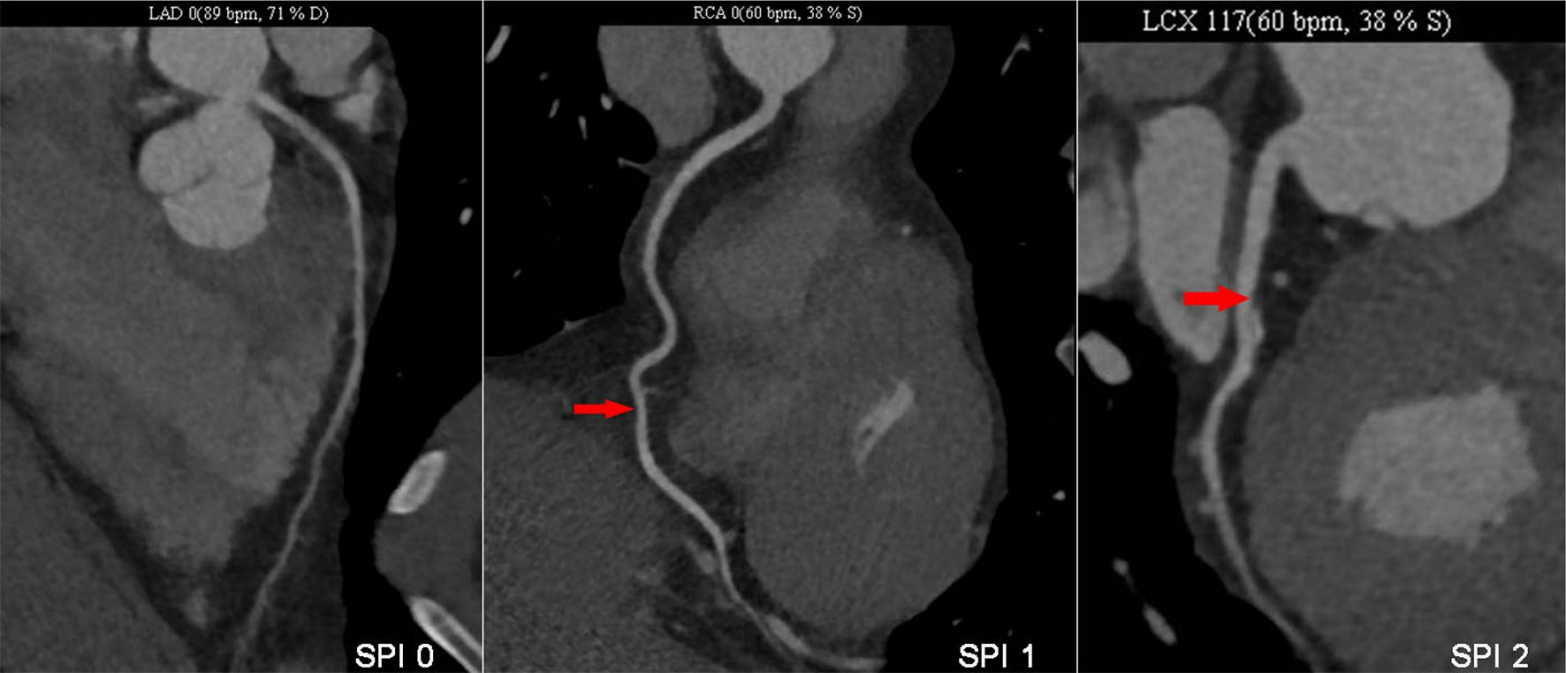
SPI classification assessed by coronary CTA.

Coronary CTA was performed according to the Society of Cardiovascular Tomography (SCCT) guidelines^7^ using the following multi-detector CT scanners: Philips Brilliance 64, Philips Medical Systems, Best, the Netherlands; Somatom Definition AS, Siemens Healthineers, Germany. Lesions on coronary CTA were then categorized based on the severity of stenosis: 0% (no CAD) and 1–49% (non-obstructive CAD). Four classifications were defined according to coronary CTA: Traditional NOCAD classification: no CAD (0% stenosis) and NOCAD (1–49% stenosis). NOCAD-RADS classification was defined according to the highest degree of coronary stenosis: NOCAD-RADS 0 (0% stenosis), NOCAD-RADS 1 (1–24% stenosis) and NOCAD-RADS 2 (25–49% stenosis). Duke prognostic NOCAD index: Duke NOCAD 0 (0% stenosis in all vessels), Duke NOCAD 1 (1–24% stenosis, or at most 1 with 25–49% stenosis) and Duke NOCAD 2 (≥ 2 vessels of 25–49% stenosis). Stenosis proximal involvement (SPI) classification: SPI 0 (no CAD, 0% stenosis), SPI 1 (1–49% stenosis with no proximal lesion) and SPI 2 (1–49% stenosis with proximal lesion) (Fig. 2). We defined proximal involvement as any plaque was present (by visual estimation) in the main or proximal segments of coronary artery (left main, left anterior descending artery, left circumflex artery, or right coronary artery).

### Follow-up and endpoint

Institutional review boards of all study centers have approved the follow-up procedures and MACE was the primary endpoint in this study. MACE was defined as all-cause death, non-fatal myocardial infarction and stroke. MACE status was determined by querying the local Community Health Service Centers. We then ascertained the event through medical records or telephone call if MACE is outside of the city. Loss of follow-up was defined as unable to obtain MACE status (without medical records or unable contacted patients by telephone outside of the city). The deadline date of follow-up was April 30, 2022.

### Statistical analysis

Continuous variables were showed as mean (± SD) and categorical variables were expressed with frequencies and percentages. One-way ANOVA test was used to compare continuous variables between groups and chi-square test was used for the comparison of categorical variables. Cumulative event-free survival was estimated by Kaplan–Meier method and log-rank test was used for comparison between groups. Hazard ratio (*HR*) with 95% confidence intervals (95% CI) was calculated by univariate and multivariate Cox proportional hazard analyses. The discriminatory values of SPI classification, traditional NOCAD classification, NOCAD-RADS classification and Duke prognostic NOCAD index for the MACE were performed by time dependent receiver-operating characteristic (ROC) curves and C-index. Clinical characteristics including sex, age, smoke, history of hypertension and diabetes and dyslipidemia were included in the multivariate Cox regression, time dependent ROC and C-index analysis. *P* < 0.05 was considered as statistically significant. All statistical analyses were carried out using R statistical package (version 4.0, R foundation for Statistical Computing, Vienna, Austria), Stata (version 16, StataCorp LP, College Station, Texas, USA) and MedCalc Statistical Software (version16.8.4 Ostend, Belgium).

## Results

Overall, the present study included 2281 (52.1%) and 2097 (47.9%) patients who had no CAD and non-obstructive CAD in the final analysis, respectively. Of the 4378 patients, 43.1% (1888 of 4378) were male and the average age was 59.3 ± 10.7 years. Table 1 presented the baseline and coronary CTA characteristics of the study population.

**Table 1:** Baseline characteristics of the study population.

	Total (N = 4378)	Survival patients (N = 4068)	MACE (N = 310)	*P*-value
Age (years)	59.3 (10.7)	58.8 (10.5)	65.9 (9.9)	< 0.001
Male gender (%)	1888 (43.1)	1714 (42.1)	174 (56.1)	< 0.001
Smoke (%)	986 (22.5)	892 (21.9)	94 (30.3)	0.001
Hypertension (%)	1919 (43.8)	1744 (42.9)	175 (56.5)	< 0.001
Diabetes (%)	791 (18.1)	720 (17.7)	71 (22.9)	0.026
Dyslipidemia (%)	1512 (34.5)	1415 (34.8)	97 (31.3)	0.239
Traditional NOCAD classification				< 0.001
0	2281 (52.1)	2166 (53.2)	115 (37.1)	
1	2097 (47.9)	1902 (46.8)	195 (62.9)	
NOCAD-RADS				< 0.001
0	2281 (52.1)	2166 (53.2)	115 (37.1)	
1	361 (8.2)	331 (8.1)	30 (9.7)	
2	1736 (39.7)	1571 (38.6)	165 (53.2)	
Duke prognostic NOCAD index				< 0.001
0	2281 (52.1)	2166 (53.2)	115 (37.1)	
1	1360 (31.1)	1250 (30.7)	110 (35.5)	
2	737 (16.8)	652 (16.0)	85 (27.4)	
SPI				< 0.001
0	2281 (52.1)	2166 (53.2)	115 (37.1)	
1	314 (7.2)	287 (7.1)	27 (8.7)	
2	1783 (40.7)	1615 (39.7)	168 (54.2)	

*SPI* stenosis proximal involvement, *NOCAD* non-obstructive coronary artery disease, *NOCAD*-*RADS* non-obstructive coronary artery disease-reporting and data system, *MACE* major adverse cardiovascular events.

**Figure 3:**
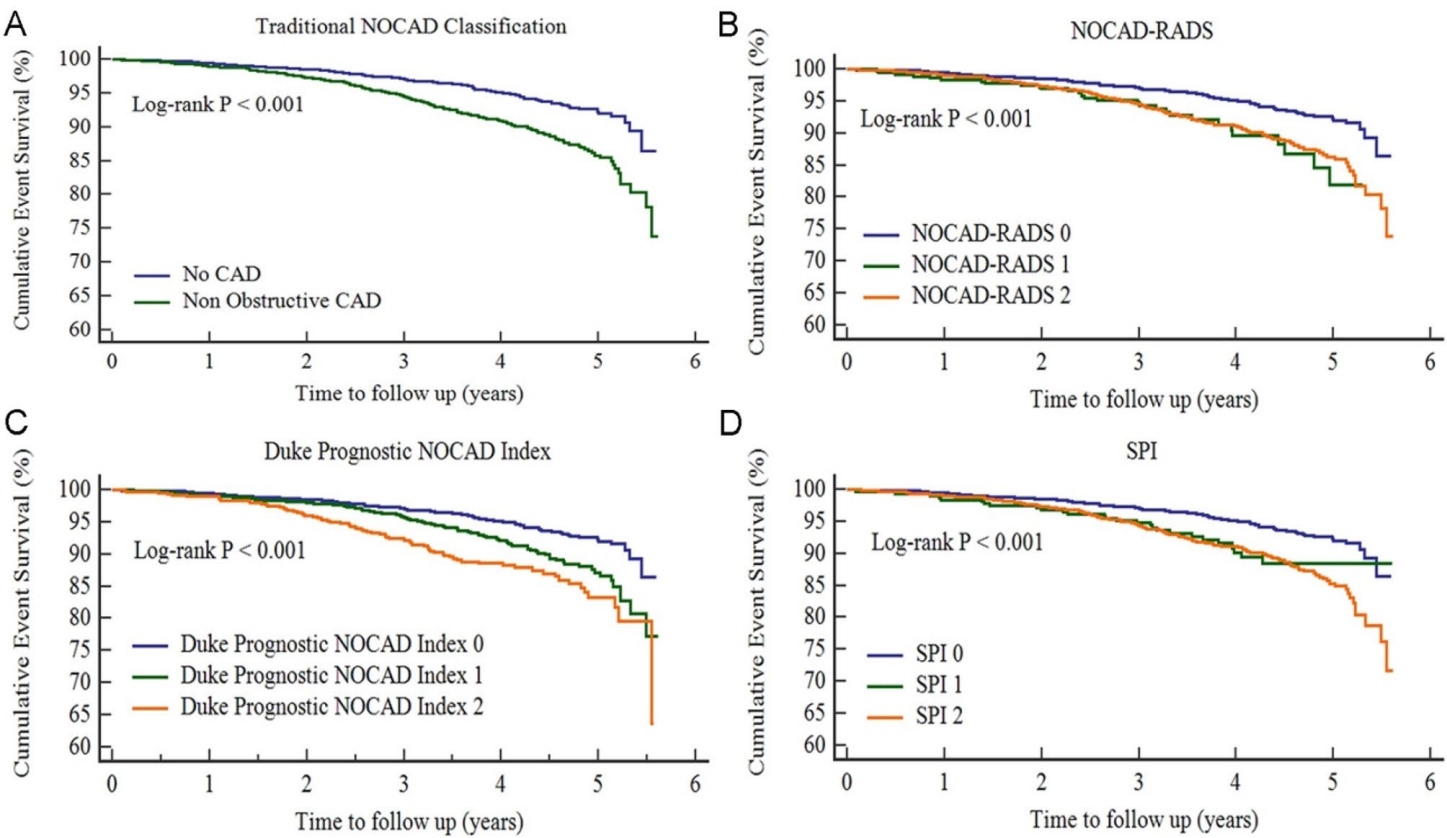
Cumulative event survivals of four classifications. *SPI* stenosis proximal involvement, *NOCAD* non-obstructive coronary artery disease, *NOCAD*-*RADS* non-obstructive coronary artery disease-reporting and data system.

In total, 310 (7.1%) death or MACE occurred during the median 3.7 years (interquartile range 3.0–4.5) of study follow-up. The annualized MACE rate was 1.36 (95% CI 1.14–1.63) and 2.52 (95% CI 2.20–2.88) for the no CAD and non-obstructive CAD (SPI 1 and SPI 2) groups, respectively (Table 2). In addition, the annualized MACE was 2.32 (95% CI 1.62–3.31) and 2.55 (95% CI 2.21–2.94) for non-obstructive CAD without proximal involvement (SPI 1) and non-obstructive CAD with proximal involvement (SPI 2), respectively after stratifying by proximal involvement for non-obstructive CAD. Kaplan–Meier survival curves indicated that the Traditional NOCAD classification, NOCAD-RADS, Duke prognostic NOCAD index, and SPI classification is significantly associated with the increasing of the cumulative events (all *P* < 0.001) (Fig. 3).

**Table 2:** Incidence of MACE.

	No. of patients	No. of MACE (%)	Annualized MACE (95% CI)
Overall	4378	310 (7.08)	1.92 (1.72–2.13)
No CAD	2281	115 (5.04)	1.36 (1.14–1.63)
Non obstructive CAD	2097	195 (9.30)	2.52 (2.20–2.88)
Without proximal involvement	314	27 (8.60)	2.32 (1.62–3.31)
With proximal involvement	1783	168 (9.42)	2.55 (2.21–2.94)

*CI* confidence intervals, *CAD* coronary artery disease, *MACE* major adverse cardiovascular events.

**Figure 4:**
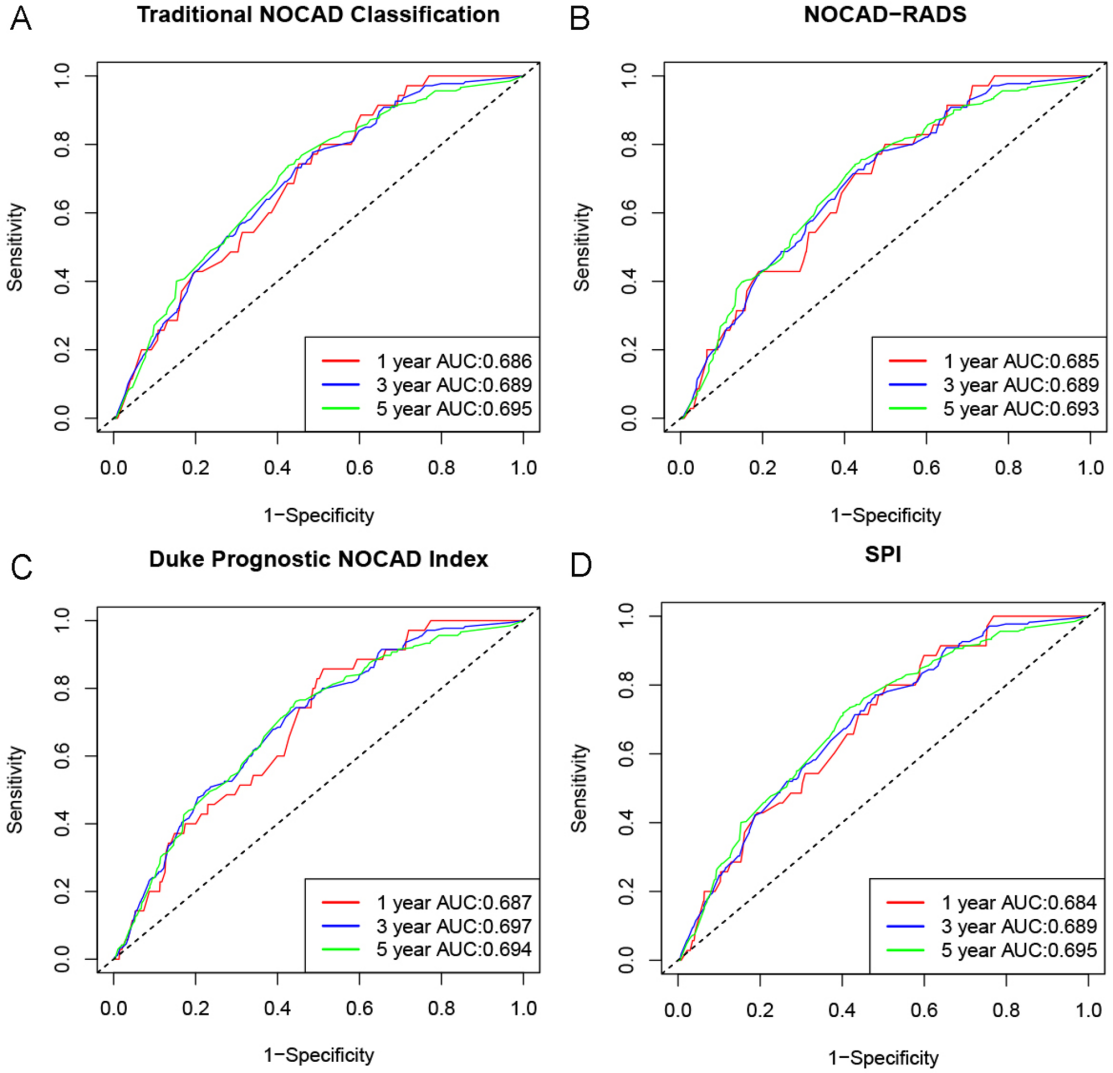
Time dependent ROC cures for prediction of MACE. *AUC* area under curve, *SPI* stenosis proximal involvement, *NOCAD* non-obstructive coronary artery disease, *NOCAD*-*RADS* non-obstructive coronary artery disease-reporting and data system.

In the univariate Cox regression analysis, SPI 1 and SPI 2 were significantly associated with MACE compared with SPI 0 (all *P* < 0.001). In multivariate Cox regressions, the risk for the MACE was increased from *HR* 1.20 (95% CI 0.78–1.83,* P* = 0.408) for SPI 1 to 1.35 (95% CI 1.05–1.73, *P* = 0.019) for SPI 2, using SPI 0 as the reference group (Table 3). The prognostic performance for predicting MACE of the classifications was using time dependent receiver-operating characteristic (ROC) curves at 1 year, 3 year and 5 year. The comparison of the prognostic performance between SPI and traditional NOCAD classification, NOCAD-RADS classification, or Duke prognostic NOCAD index was present in Fig. 4. The area under the time dependent ROC curve (AUC) for prediction of MACE was 0.684, 0.689, 0.695 for SPI classification in 1 year, 3 year, 5 year, respectively, which was similar with the results of Traditional NOCAD classification, NOCAD-RADS classification and Duke prognostic NOCAD index. In addition, adding SPI as a predictor to models (adjustment with clinical characteristics) did not improved their predictive value for MACE (Table 4).

**Table 3:** Baseline characteristics and coronary CTA findings associated with MACE.

	Univariable HR (95% CI)	P-value	Multivariable HR (95% CI)	P-value
Age (years)				
< 60	Reference		Reference	
≥ 60	2.78 (2.16–3.58)	< 0.001	2.58 (2.00–3.36)	< 0.001
Male gender	1.77 (1.41–2.21)	< 0.001	1.68 (1.29–2.19)	< 0.001
Smoke	1.52 (1.20–1.94)	0.001	1.18 (0.88–1.56)	0.269
Hypertension	1.76 (1.40–2.20)	< 0.001	1.37 (1.08–1.73)	0.009
Diabetes	1.34 (1.03–1.75)	0.030	1.11 (0.84–1.45)	0.462
Dyslipidemia	0.90 (0.71–1.15)	0.400	0.93 (0.73–1.19)	0.560
SPI				
0	Reference		Reference	
1	1.69 (1.11–2.57)	0.014	1.20 (0.78–1.83)	0.408
2	1.95 (1.54–2.47)	< 0.001	1.35 (1.05–1.73)	0.019

*SPI* stenosis proximal involvement, *HR* hazard ratios, *CI* confidence intervals, *MACE* major adverse cardiovascular events.

**Table 4:** Performance and comparison of predictive models for MACE.

Model*	C-index	*P*-value
Traditional NOCAD classification	0.679	
Traditional NOCAD classification + SPI	0.682	0.961^#^
NOCAD-RADS	0.680	
NOCAD-RADS + SPI	0.681	0.952^&^
Duke prognostic NOCAD index	0.685	
Duke prognostic NOCAD index + SPI	0.687	0.897^^^

*SPI* stenosis proximal involvement, *NOCAD* non-obstructive coronary artery disease, *NOCAD*-*RADS* non-obstructive coronary artery disease-reporting and data system, *MACE* major adverse cardiovascular events.

*Adjustment with sex, age, smoke, hypertension, diabetes and dyslipidemia.

^#^When compared traditional NOCAD classification with traditional NOCAD classification + SPI.

^&^When compared NOCAD-RADS with NOCAD-RADS + SPI.

^^^When compared Duke prognostic NOCAD index with Duke prognostic NOCAD index + SPI.

## Discussion

The current study demonstrated that the novel SPI classification had prognostic value for the prediction of MACE among patients with suspected no obstructive CAD in a retrospective, observational, multicentre study in the Chinese population, which was non-inferior to traditional NOCAD classification, NOCAD-RADS classification and Duke prognostic NOCAD index. Moreover, the presence of non-obstructive plaque in proximal coronary segments was associated with a 1.35-fold higher risk of MACE compared to patients with no CAD as assessed by coronary CTA. In addition, the mid or distal segments involvement in patients with non-obstructive CAD was not associated with the increasing of MACE risk compared to patients without CAD. These findings suggested that evaluating the location of coronary plaque in coronary CTA images may improve the practicability of coronary CTA in risk stratification of patients with non-obstructive CAD.

Previous studies have revealed that proximal coronary segments place higher weights on contribution to the total left ventricular blood flow and the volume affected of myocardium^8,10,14^. Moreover, plaque rupture and thrombus occlusion have been demonstrated to be more likely to occur in the proximal third of the coronary arteries in previous angiographic studies^15,16^. In addition, accumulating evidence has shown that the presence and severity of CAD in the proximal coronary segments are closely associated with poor prognosis^10,11,17,18^. However, these studies mainly focused on the prognostic significance of proximal plaque involvement in obstructive CAD. The contribution of proximal involvement to MACE in patients with non-obstructive is not well established.

Our previous study has showed that the prevalence of non-obstructive CAD is higher than that of obstructive CAD^13^, which is consistence with other multicenter studies^4,5^. Moreover, recent studies have suggested that the majority of cardiovascular events and all-cause mortality occurred among patients with non-obstructive CAD^5,13^. Thus, it is necessary to provide further risk stratification for the evaluation and management of non-obstructive CAD. Though traditional non-obstructive CAD classification presented a significant increased risk of MACE for non-obstructive CAD compared with no CAD, as shown in the present study. This classification lacks further risk stratification for patients with non-obstructive CAD, which may lead to overtreatment and management of this population. NOCAD-RADS and Duke prognostic NOCAD index classification provide more detailed risk stratification than that of traditional non-obstructive CAD classification^10,13,19,20^. This is in line with our findings. However, risk stratification in these studies was based on coronary stenosis rather than stenosis proximal involvement assessed by coronary CTA. Other study revealed that risk stratification improvement of non-obstructive CAD can be characterized by the extent of affected coronary segments evaluated by coronary CTA^21^. The few studies that do focus stenosis proximal involvement on risk stratification in patients with non-obstructive CAD. The recent CONFIRM registry study showed that proximal non-obstructive CAD had greater risk of MACE compared to patients with no CAD^12^. Our findings confirmed and expand these previous findings by demonstrating the proximal involvement was independently associated with increased MACE in of patients with non-obstructive CAD in Chinese population. Moreover, we compare the prognostic value of SPI with existed classifications and showed non-inferior to traditional NOCAD classification, NOCAD-RADS classification and Duke prognostic NOCAD index. Furthermore, considering both degree of stenosis and proximal involvement of CAD, the risk stratification of patients with non-obstructive CAD was improved.

Despite the import findings and clinical implications for SPI prognostic value in patients with suspected CAD in the present study, the study had several limitations. First, the study contains a relative larger sample size; however, the selection bias may be present with the retrospective nature of this study. Second, the numbers of classes are different according to the classifications that may lead inconsistent of the proportion between complex classifications and simple classifications. Specially, the relatively small sample size of patients in SPI 1 group may lead to inadequate detection of prognostic differences based on proximal involvement in non-obstructive CAD. Larger samples and multicenter researches are needed to reduce bias. Third, due to the unavailability of the data on specific causes of death, the clinical endpoint was MACE, defined as all-cause mortality, myocardial infraction or stroke. Cardiac mortality could not be separately assessed as an additional outcome which would be expected to have a stronger correlation with atherosclerotic burden. Finally, the present study had limited data on coronary artery calcium (CAC) which was recently shown to be a predictor of risk for death in non-obstructive CAD^22^.

In conclusion, Coronary CTA based SPI classification provided important prognostic information for MACE risk prediction in patients with non-obstructive CAD, which was non-inferior than traditional NOCAD, Duke NOCAD index and NOCAD-RADS classifications. The plaque location information by coronary CTA may provide additional risk prediction in patients with non-obstructive CAD.

## Data Availability

The datasets generated during and analyzed during the current study are available from the corresponding author on reasonable request.

## Abbreviations

CVD: Cardiovascular disease
CAD: Coronary artery disease
CONFIRM: Coronary CT angiography evaluation for clinical outcomes: an international multicenter
CTA: Computed tomography angiography
MACE: Major adverse cardiovascular events
SCCT: Society of cardiovascular tomography
SPI: Stenosis proximal involvement
NOCAD: Non-obstructive coronary artery disease
NOCAD-RADS: Non-obstructive coronary artery disease-reporting and data system
HR: Hazard ratios
CI: Confidence intervals
